# Ultrawide Bandwidth Electromagnetic Wave Absorbers Using a High-capacitive Folded Spiral Frequency Selective Surface in a Multilayer Structure

**DOI:** 10.1038/s41598-019-52967-z

**Published:** 2019-11-11

**Authors:** Tian Liu, Sung-Soo Kim

**Affiliations:** Department of Advanced Materials Engineering, Chungbuk National University, Cheongju, 28644 Korea

**Keywords:** Electronic properties and materials, Electrical and electronic engineering

## Abstract

A high-capacitive frequency selective surface (FSS) with a new structure of folded spiral conductors is proposed as the small-array periodicity and low-frequency resonance FSS for ultra-wide bandwidth absorbers in a multilayer structure. Due to the folded structure with long effective segments and a small gap, a large value of capacitance for the lowest resonating frequency is obtained. Through a combination of the high-capacitive spiral FSS with other conventional FSSs (square loop, square patch) with a medium- and high-frequency resonance, an ultra-wide absorption bandwidth (4.7–50.0 GHz for −10 dB reflection loss) is designed with a small total thickness of 7.0 mm, which is close to the theoretical limit (6.7 mm). Admittance analysis is conducted for better insight into the optimization procedure. The free space measurement with a test sample prepared by the screen printing method also demonstrates a wide-bandwidth absorption result (5.2–44.0 GHz for −10 dB reflection loss, total thickness = 6.5 mm), which is in good agreement with the simulation result. In addition, the angular stability of the proposed wide-bandwidth absorber is discussed for both TE and TM polarizations in association with unit cell periodicity and grating lobes.

## Introduction

The electromagnetic radiation from diverse electronics of high-speed digital and analogue circuits over the broad frequency spectrum is considered a critical challenge regarding the electromagnetic interference, compatibility, and reliability of electronic systems. For example, wireless communication systems, including local area networks (LANs) and the Internet of Things (IoT), using fifth-generation (5 G) wireless technology, which will offer a much faster, scalable and efficient network, have found a wide range of applications during the past few decades or will do in the near future^1^. High-performance electromagnetic wave absorbers are required for electromagnetic wave control to eliminate interference between electronic devices and systems, particularly with a broad bandwidth from radio frequency to a millimeter wave spectrum, which will play a big role in future 5 G wireless networks. Unfortunately, there is a theoretical barrier in widening the absorption bandwidth at the given conditions of wave reflectance and the thickness of the absorber because of an inverse proportional relationship among those performance parameters^2^.

The figure of merit on absorber performance can be represented by the fractional bandwidth (FBW), which is defined by the ratio of the bandwidth to the center frequency with respect to a certain absorption level, and by the layer thickness expressed in wavelength at the lowest operation frequency (λ_L_). The conventional narrow-band absorbers, such as Salisbury screens^3^ or Dallenbach layers^4^, exhibited a single absorption peak with a typical value of the FBW smaller than 20%. Recently proposed metamaterial absorbers exhibited a much narrower bandwidth due to their resonating characteristics, even though they had the advantage of an extremely small thickness^5–7^. There have been many approaches to widen the bandwidth; one constructs a multilayered structure and another uses frequency selective surfaces (FSSs). The Jaumann absorber^8–10^ is one example of a broad bandwidth absorber with a multilayer structure with a very large FBW up to 170%, but it suffers from a much higher thickness (∼0.3λ_L_). The use of frequency selective surfaces^11–18^ or metasurfaces^19–22^ on a nonmagnetic substrate is an effective approach to widen the bandwidth while keeping a small thickness. For example, an experimental result for a 10 dB absorption bandwidth of 5.3–18.0 GHz (FBW = 108%) was reported with a 4 mm (0.07λ_L_) thick absorber using an FSS with a crisscross and fractal square patch^15^.

Further enhancement of absorption bandwidth has been realized using multilayers of FSSs or metasurfaces^23–30^. A multilayer absorber composed of four layers of split ring resonators (SRRs) with different geometries has been proposed to realize a 90% absorptive bandwidth of 10–70 GHz (FBW = 150%) with a somewhat large overall thickness (∼0.15λ_L_)^25^. In our previous study^30^, through a layer combination of two FSS patterns with different resonating frequencies, an ultra-wide absorption bandwidth (6.3–40.0 GHz (FBW = 145%) for −10 dB reflection loss) was designed with a small total thickness of 5.5 mm (0.11λ_L_). Summarizing the state of the art in existing studies on broad bandwidth absorbers of multilayered FSS structures, the top results were at the level of FBW = 140–150% with a total thickness = 0.10–0.15λ_L_.

In the ultra-wide bandwidth FSS absorbers, the bandwidth is limited by the lowest frequency (*f*_L_), which is a theoretical value determined from the given reflectance and thickness^2^. The highest absorption frequency (*f*_H_) is limited by the array structure of an absorber in relation to the grating lobes or the harmonic waves of fundamental resonance^30,31^. Rozanov^2^ demonstrated a physical bound on the lowest achievable frequency of an absorber, which is given by *f*_L_ = cΓ_0_/172*d* for a multilayered non-magnetic absorber, where c is the velocity of light, Γ_0_ is the reflection coefficient in dB, and *d* is the total thickness of the absorber. The larger the reflectance or the smaller the thickness, the higher the lowest frequency (i.e., the bandwidth is decreased). This frequency is inversely proportional to the spatial periodicity of the FSS. Consequently, increasing the spatial periodicity is a simple solution for increasing the lowest bound. However, this effort will decrease the high frequency bound, which is limited by the grating lobes or high-frequency harmonics of fundamental resonance^30,31^. The first factor limiting the high frequency bandwidth is the excitation of grating lobes at frequencies higher than the lowest cutoff frequency (Floquet’s modes), which is also inversely proportional to the spatial periodicity of the FSS^31,32^. Consequently, it might seem that decreasing the spatial periodicity is a simple solution for increasing *f*_H_. Unfortunately, a decrease in periodicity results in a decrease in inductance and capacitance of an FSS and thus an increase in the lowest frequency *f*_L_. Therefore, the minimum possible periodicity with a low resonating frequency as well should be required for the design of ultra-wide bandwidth absorbers, which can be accomplished by using an FSS with large circuit parameters (inductance, capacitance).

Linear and crossed dipoles are the simplest of element designs used in FSS arrays. Convoluting the conductor or wrapping it in a spiral configuration is clearly capable of lowering the resonant frequency while leaving the array periodicity unaltered^33–37^. Alternatively, the unit-cell size needed for operation at a given frequency can be efficiently reduced. Such a dramatic decrease in resonance frequency in the interwoven crossed dipoles or entwined spirals is due to the large equivalent capacitance^37^, which is due to the coupling between the intertwined spiral conductors distributed over their entire length. It adds up and increases the total unit cell capacitance, which is a very effective method for lowering the resonant frequency with the array periodicity unaltered, and can be utilized in the design of ultra-wide bandwidth absorbers.

Here we propose a high-capacitive FSS with a new structure of four folded arm spiral conductors to achieve an ultra-wide bandwidth absorber using a small-array periodicity and low-frequency resonance FSS. Due to the folded structure with long effective segments and a small gap, a large capacitance value for the lowest resonating frequency was obtained. Through a combination of the high-capacitive spiral FSS with other conventional FSSs (square loop, square patch) with a medium- and high-frequency resonance, we designed an ultra-wide absorption bandwidth (4.7–50.0 GHz for −10 dB reflection loss) with a small total thickness of 7.0 mm, which is close to the theoretical limit. That is the novelty of this study over the existing studies for enlarged bandwidth (fractional bandwidth 166%) with a comparable thickness (0.11λ_L_) and a simple and polarization-insensitive structure. The free space measurement with a test sample prepared by the screen printing method also demonstrated a wide-bandwidth absorption result (5.2–44.0 GHz (FBW = 158%), total thickness = 6.5 mm (0.12λ_L_)), and it was in good agreement with the simulation result. In addition, we dealt with the angular stability of the proposed wide bandwidth absorber for both TE and TM polarization in association with unit cell periodicity and grating lobes.

## Results

### Inductance and capacitance of FSSs

**Figure 1:**
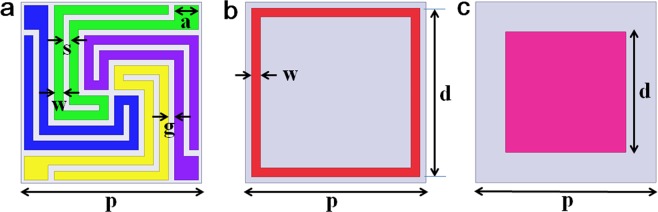
Schematic of the three types of FSSs and their dimensions: (**a**) spiral FSS (p = 6.0 mm, w = 0.3 mm, s = 0.2 mm, g = 0.2 mm, a = 0.8 mm), (**a**) square loop FSS (p = 6.0 mm, d = 5.6 mm, w = 0.3 mm), and (**c**) square patch FSS (p = 6.0 mm, d = 4.0 mm).

Figure 1 illustrates the three types of FSS (spiral, square loop, square patch) and their dimensions. In the spiral FSS (designated S-FSS), double-folded strips of width (w = 0.3 mm) and gap (s = 0.2 mm) was convoluted in a clockwise spiral configuration with three turns (Fig. 1a). The strip terminated at the corner as a square patch with the size a = 0.8 mm. A unit cell (with a periodicity p = 6 mm) was filled with four arms of the spiral strips with 90° rotation symmetry and was thus polarization insensitive. The spacing between adjacent folded strips was g = 0.2 mm, which was uniform throughout the unit cell. For the square loop FSS (designated SL-FSS), the dimensions were the unit cell period p = 6.0 mm, the FSS length d = 5.6 mm, and the FSS width w = 0.3 mm (Fig. 1b). The square patch FSS (designated SP-FSS) were p = 6.0 mm, d = 4.0 mm (Fig. 1c).

**Figure 2:**
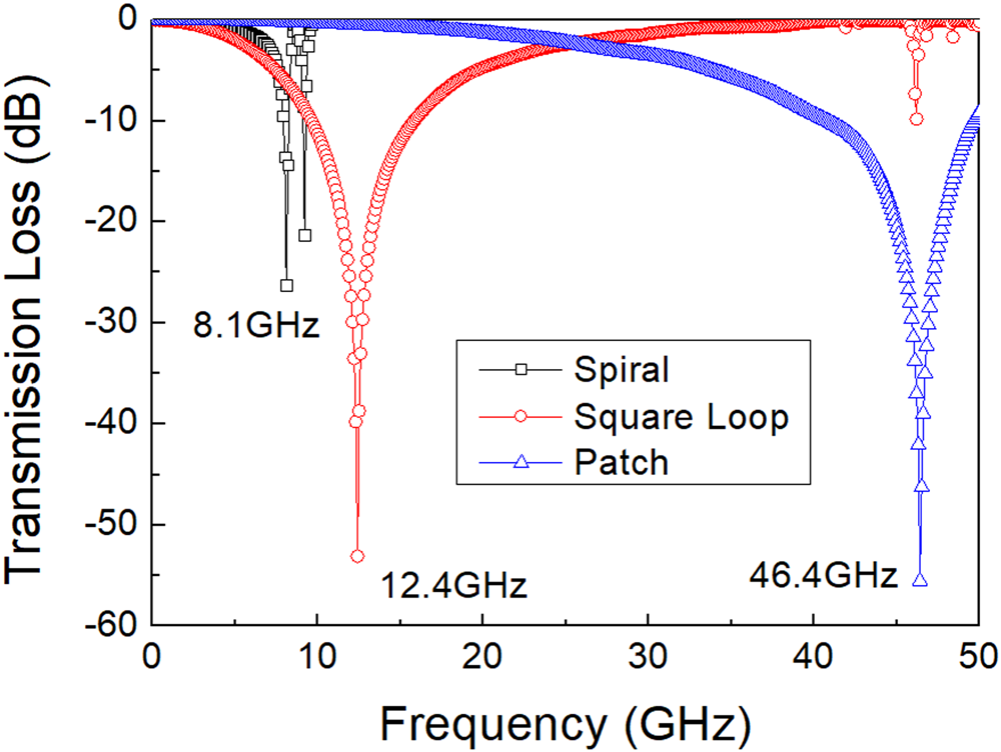
Simulation results of the transmission loss for the three types of FSS (spiral, square loop, square patch) standing in free space with the dimensions given in Fig. 1.

A unit cell of the FSS may be represented as a transmission line loaded with a shunt reactance composed of inductance *L* and capacitance *C* connected in series. The FSS conductors were assumed to be perfect conductors (i.e. resistance *R* is zero). Figure 2 presents the simulation result in which this array had a principal resonance at a frequency (*f*_0_) of 8.1 GHz for S-FSS, 12.4 GHz for SL-FSS, and 46.4 GHz for SP-FSS. For the S-FSS and SL-FSS, the inductance and capacitance were calculated from the equations of reactance and susceptance at the resonance frequency of the equivalent circuit^17,38–40^ and the results were *L* = 1.16 nH and *C* = 288.46 fF for S-FSS and *L* = 2.77 nH and *C* = 90.62 fF for SL-FSS. For the S-FSS, a large capacitance (more than three times that of the SL-FSS with the same periodicity) was predicted, which arose from the inter- and intra-coupling between the folded spiral conductors distributed over a large effective length with a small spacing^40^. However, the value of the total equivalent inductance was smaller than that in the SL-FSS due to the parallel circuit connection of the shunted inductance of each arm^40^. For the SP-FSS, applying the strip wire conductor model^41^ revealed *L* = 0.31 nH and *C* = 35.40 fF, both of which were very low compared with the S-FSS or SL-FSS, and thus high-frequency resonance was achieved. Table 1 summarizes the results. The resonance frequency calculated from the *L* and *C* values was *f*_0_ = 8.7 GHz for S-FSS, *f*_0_ = 12.4 GHz for SL-FSS, and *f*_0_ = 46.1 GHz for SP-FSS, which was consistent with the simulation results presented in Fig. 2. The quarter wavelength (λ/4) at the resonance frequency is also given in Table 1.

**Table 1:** Circuit parameters (*L*, *C*), resonance frequencies (*f*_0_), and quarter wavelengths (λ/4) at *f*_0_ for three types of FSS.

Pattern	*L* nH	*C* fF	*f*_0_ (calc.) GHz	*f*_0_ (sim.) GHz	λ/4 mm
Spiral	1.16	288.46	8.7	8.1	9.3
Square Loop	2.77	90.62	12.4	12.4	6.0
Square Patch	0.31	35.40	46.1	46.4	1.6

### Single-layer FSS absorber design

**Figure 3:**
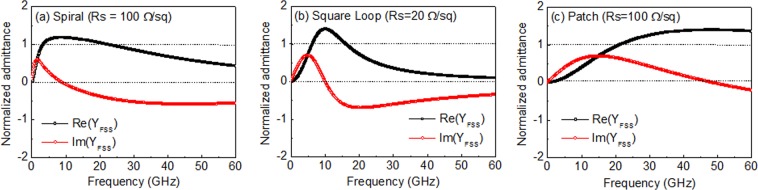
Normalized admittance (Y_FSS_/Y_0_) of the resistive FSSs: (**a**) S-FSS (*R*_s_ = 100 Ω/sq), (**b**) SL-FSS (*R*_s_ = 20 Ω/sq), and (**c**) SP-FSS (*R*_s_ = 100 Ω/sq).

Figure 3 illustrates the normalized admittance of the FSS (Y_FSS_/Y_0_ = Re(Y_FSS_) + jIm(Y_FSS_), where Y_0_ is free-space admittance), which was derived from the circuit parameters (*R*, *L*, *C*). The FSS resistance (*R*) had a relationship with the surface resistance of the conductor (*R*_s_); *R* ≈ *R*_s_(B/A), where A is the effective area of the conductors (for example, A = d × d for SP-FSS) and B is the unit cell area (= p × p). Supplementary Table S1 presents the *R* values of the FSS with an increasing surface resistance. The imaginary part of the normalized admittance was zero (Im(Y_FSS_) = 0) at the resonance frequency determined from *f*_0_ = 1/2π(*LC*)^1/2^. With respect to the resonance frequency, the circuit was capacitive (Im(Y_FSS_) ≥ 0) at lower frequencies and inductive (Im(Y_FSS_) ≤ 0) at higher frequencies. This circuit behavior of the FSS exhibited an inverse frequency dispersion at the high-impedance surface of the grounded substrate with a λ/4 thickness, and thus could be cancelled out around the resonance frequency. The real part Re(Y_FSS_) exhibited a broad dispersion due to the resistive component of the FSS. The FSS resistance approached a free-space impedance (377 Ω) at the given values of *R*_s_ = 118, 35, 85 Ω/sq for the S-FSS, SL-FSS, and SP-FSS, respectively.

**Figure 4:**
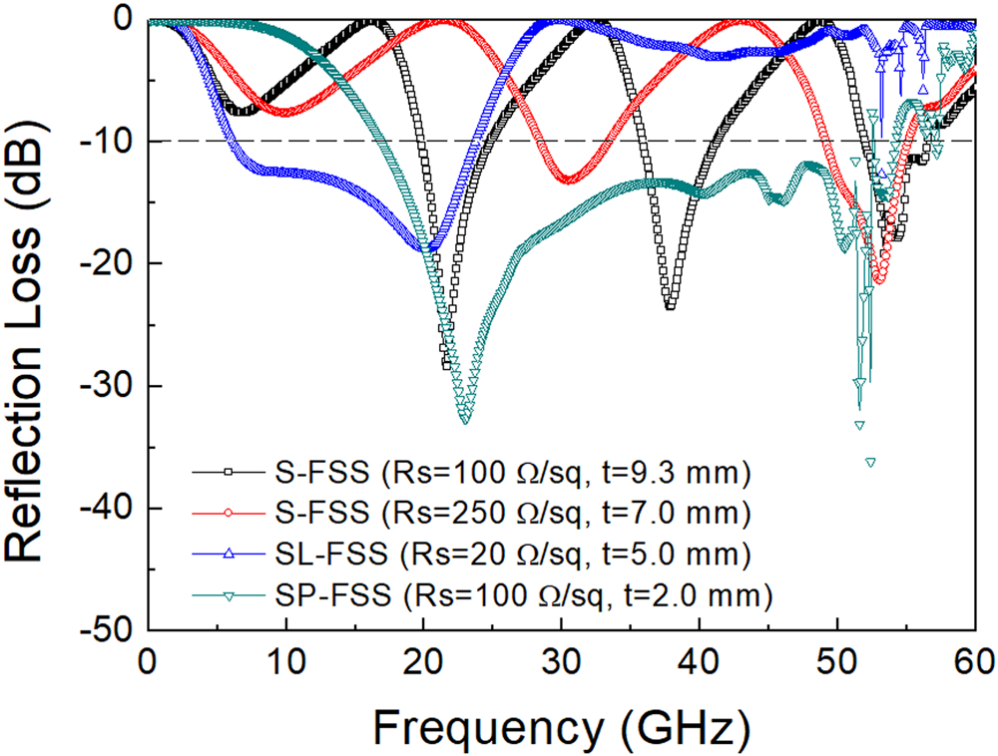
Reflection loss determined for the single-layer FSS absorber with a spacer thickness (t) and surface resistance (*R*_*s*_) indicated inside the figure.

In this way, a single-layer microwave absorber with a broad bandwidth could be designed with the optimized FSS on the surface of the grounded substrate. For simplicity, the substrate material was assumed to be air and we used the *L* and *C* values of the FSS as determined previously. Figure 4 presents the reflection loss for the single-layer FSS absorbers designed with an optimized admittance matching combination of the FSS and the substrate for normal incidence of TEM wave. For the S-FSS (*R*_s_ = 100 Ω/sq which corresponds to circuit resistance *R* = 320 Ω) on a grounded air substrate with a λ/4 thickness (t = 9.3 mm), multiple absorption peaks were observed at frequencies of 6.8 GHz, 21.6 GHz, 37.8 GHz, and 40.4 GHz, which resulted from the dimensional resonance of the grounded substrate (Supplementary Fig. S1a). When the thickness was reduced (t = 7 mm), the absorption peaks shifted to higher frequencies (9.8 GHz, 30.5 GHz, and 53.0 GHz) due to a smaller substrate thickness of a short-circuit resonance (Supplementary Fig. S1b). For the SL-FSS (*R*_s_ = 20 Ω/sq corresponding to *R* = 214 Ω) on a grounded air substrate (t = 5.0 mm), a broad bandwidth was predicted with a reflection loss lower than –10 dB in the frequency range of 6.1–23.4 GHz. For the SP-FSS (*R*_s_ = 100 Ω/sq corresponding to *R* = 225 Ω, and t = 2.0 mm), a broad bandwidth at high frequencies was predicted with a reflection loss lower than –10 dB in the high frequency range 18.5–52.4 GHz. Many small peaks of reflection were observed above 50 GHz due to the grating lobes or high frequency harmonics, which occurred in the high frequency region above which the unit cell period (p = 6 mm) was larger than the wavelength (p ≥ λ)^31,32^.

### Multi-layer FSS absorber design

**Figure 5:**
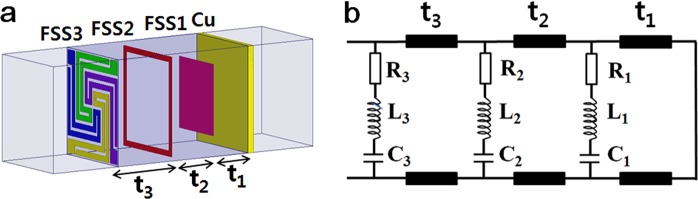
Illustration of (**a**) triple-layered FSS absorbers with a combination of FSS1/FSS2/FSS3 (square patch/square loop/spiral) and (**b**) equivalent circuit.

Next a multilayer electromagnetic absorber with a wide bandwidth was designed with a combination of the three resistive FSS layers, as illustrated in Fig. 5a. The operation of the FSS absorber is explained using the equivalent circuit presented in Fig. 5b. For the triple-layer FSS absorbers, FSS1 (square patch) was placed on a grounded substrate with a thickness of t_1_, FSS2 (square loop) was placed on the second substrate with a thickness of t_2_, and FSS3 (spiral) was placed on the third substrate with a thickness of t_3_, which was in the order of increased λ/4 thickness from the ground plane. The substrate was assumed to be air in order to use the *L* and *C* values of the FSS determined previously. In the triple-layer FSS absorber, however, multiple reflections occurred at the three FSSs, especially at a band in which triple absorption peaks of each single-layer absorber overlapped, so optimization of the design parameters of the FSS (surface resistance of FSS and spacer thickness) was required for the widest bandwidth.

**Figure 6:**
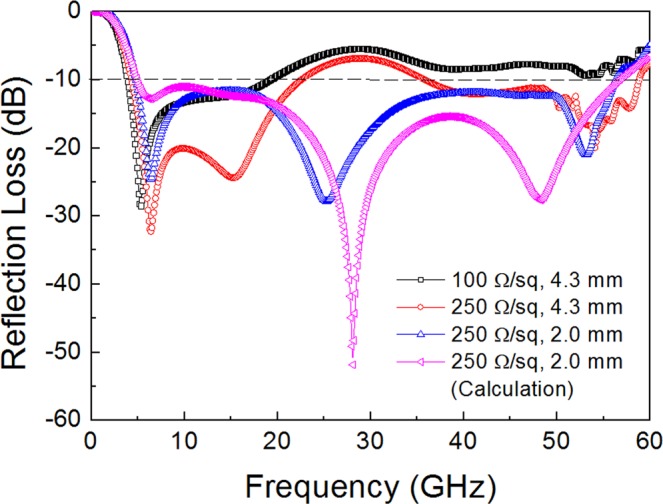
Reflection loss determined in the triple-layer FSS absorber with optimizing the surface resistance and the substrate thickness of FSS3 (*R*_s3_ = 100∼250 Ω/sq, *t*_3_ = 2.0∼4.3 mm). The other parameters of FSS1 (SP-FSS with *R*_s1_ = 100 Ω/sq and *t*_1_ = 2.0 mm) and FSS2 (SL-FSS with *R*_s2_ = 20 Ω/sq and *t*_2_ = 3.0 mm) are fixed. The calculated result using circuit parameters is also given for comparison.

Figure 6 presents the simulated reflection loss determined in the triple-layer FSS absorber with optimizing the surface resistance of FSS3 (*R*_s3_ = 100–250 Ω/sq) and a substrate thickness of FSS3 (t_3_ = 2.0–4.3 mm) for normal incidence of TEM wave. The other parameters of FSS1 (SP-FSS with *R*_s1_ = 100 Ω/sq and t_1_ = 2.0 mm) and FSS2 (SL-FSS with *R*_s2_ = 20 Ω/sq and t_2_ = 3.0 mm) were fixed. With a layer structure of a simple combination of the optimized single-layer FSS (FSS1 with SP-FSS (*R*_s1_ = 100 Ω/sq, t_1_ = 2.0 mm), FSS2 with SL-FSS (*R*_s2_ = 20 Ω/sq, t_2_ = 3.0 mm), and FSS3 with S-FSS (*R*_s3_ = 100 Ω/sq, t_3_ = 4.3 mm)), the reflection loss was higher than −10 dB in the frequencies above 19.2 GHz. Increasing the surface resistance to *R*_s3_ = 250 Ω/sq (corresponding to circuit resistance *R*_3_ = 825 Ω) has the effect of lowering the reflection loss across all the frequencies. However, the reflection loss was still higher than −10 dB in the middle frequency region of 22.0–35.0 GHz. Reducing the substrate thickness of FSS3 (t_3_ = 2.0 mm) further enhanced the microwave absorption, producing a 10 dB absorption bandwidth of 4.7–56.4 GHz, as depicted in Fig. 6. A good agreement is noted when comparing the results on the reflection loss and 10 dB bandwidth simulated using HFSS and calculated using circuit parameters.

Table 2 summarizes the optimized design parameters for the triple-layer FSS absorbers with the widest bandwidth. At the values *R*_s1_ = 100 Ω/sq (t_1_ = 2.0 mm), *R*_s2_ = 20 Ω/sq (t_2_ = 3.0 mm), *R*_s3_ = 250 Ω/sq (t_3_ = 2.0 mm), the reflection loss decreased to less than –10 dB in the frequency range of 4.7–56.4 GHz. The fractional bandwidth approached the maximum value, FBW = 170%. To our best knowledge, this is the first demonstration for the widest bandwidth absorber. Even if we take the highest frequency limit as the critical frequency of grating lobes at the given array periodicity (∼50 GHz), the fractional bandwidth is still very broad (FBW = 166%) with respect to a −10 dB reflection loss. The total thickness was 7 mm, equal to 0.11λ_L_, and very close to the theoretical limit (6.7 mm) determined from Rozanov’s equation^2,28^. It is evident that the bandwidth of the triple-layer absorber was maximized through a combination of the spiral FSS with the lowest resonating frequency with two other SL-FSS and SP-FSS whose resonance frequency was so sufficiently separated that their original absorption bands did not much overlap.

**Table 2:** Optimized design parameters for triple-layer absorbers with a FSS1/FSS2/FSS3 (square patch/square loop/spiral) combination depicting the widest bandwidth.

FSS	*R*_s_ Ω/sq	*L* nH	*C* fF	t mm
FSS1 (Patch)	100	0.31	35.4	2.0
FSS2 (Loop)	20	2,96	83.0	3.0
FSS3 (Spiral)	250	1.16	288.5	2.0

**Figure 7:**
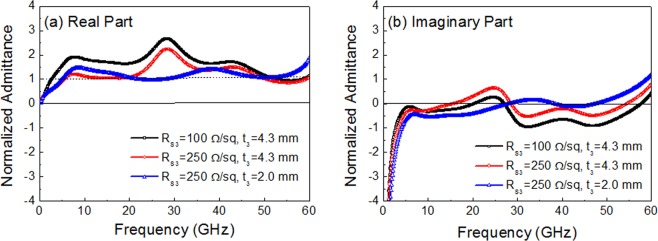
Normalized admittance by free space admittance of triple-layer absorber with varying the surface resistance of FSS3 (*R*_s3_ = 100∼250 Ω/sq) and the substrate thickness of FSS3 (t_3_ = 2.0∼4.3 mm) at a fixed other parameters of FSS1 (SP-FSS with *R*_s1_ = 100 Ω/sq and t_1_ = 2.0 mm) and FSS2 (SL-FSS with *R*_s2_ = 20 Ω/sq and t_2_ = 3.0 mm): (**a**) real part and (**b**) imaginary part.

The admittance analysis gave us a better insight into the effect of the surface resistance of the FSS and the substrate thickness on the reflection loss and bandwidth of the triple-layer absorber. Figure 7 presents the real and imaginary part of total admittance normalized by free-space admittance at the surface of the triple-layer absorber calculated from the equivalent circuit. For the original design with *R*_s1_ = 100 Ω/sq (t_1_ = 2.0 mm), *R*_s2_ = 20 Ω/sq (t_2_ = 3.0 mm), *R*_s3_ = 100 Ω/sq (t_3_ = 4.3 mm), the real part of admittance was much higher than the matching value in most of the frequencies (Fig. 7a), which caused the higher reflection loss. Surface resistance needed to be raised to suppress the real part of admittance. This optimization was the most effective for FSS3 (S-FSS), since it had a high capacitance and the lowest resonance frequency and thus a high value of the real part of admittance in a broad frequency range (Fig. 3a). By increasing the surface resistance of S-FSS from *R*_s3_ = 100 Ω/sq to *R*_s3_ = 250 Ω/sq, the real part of admittance was decreased (Fig. 7a), and the absorption properties were improved (Fig. 6).

Another critical design parameter for the bandwidth was the spacer thickness, particularly for FSS3 (t_3_). Reduction from t_3_ = 4.3 mm to t_3_ = 2.0 mm lowered the reflection loss particularly in the middle frequency region (22.0–35.0 GHz), due to the readjusted coupling between FSSs toward a new admittance matching. The enlargement of the inductive frequency region in that middle frequency region with a decreasing substrate thickness (Supplementary Fig. S1b) had the effect of moving the absorption band to higher frequencies, as seen in the imaginary part of admittance (Fig. 7b) and the absorption curve of the single-layer S-FSS (Fig. 4). This frequency response counteracted the intrinsic capacitive behavior of the FSS, particularly for FSS1 (patch), which made it possible to approach admittance matching in the middle frequency region of strong coupling, as clearly seen in both real and imaginary parts of admittance (Fig. 7).

**Figure 8:**
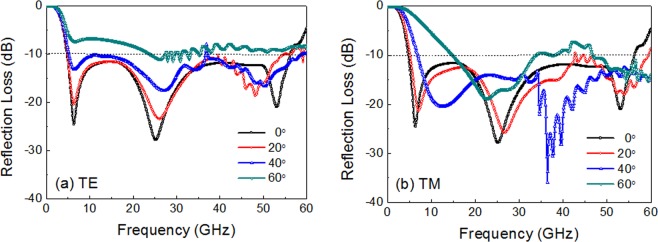
Simulation results of reflection loss of the triple-layer FSS absorber with an increasing incidence angle for (**a**) TE and (**b**) TM polarization.

The angular stability of the proposed wide bandwidth absorber was investigated over wide incidence angles for both TE and TM polarization. For TE polarization, the reflection loss increased as the incidence angle (θ) increased, but the 10 dB absorption bandwidth was maintained up to 40° (Fig. 8a). For TM polarization, the bandwidth decreased at high incidence angles above 40° (Fig. 8b). The grating lobes or high frequency harmonics (indicated by serration in the reflection loss curves) were observed in the high frequency region, and the critical frequency decreased with increasing incidence angles for both TE and TM polarization. For θ = 40°, the critical frequency decreased to as low as 37 GHz (for TE) and 34 GHz (for TM), approximately following the equation p ≤ λ/(1 + sinθ)^42^. In comparison with a previous study^30^, however, the angular stability concerning the bandwidth and the critical frequency of grating lobes was greatly improved, which was due to the decreased unit cell periodicity.

### Experimental verification

**Figure 9:**
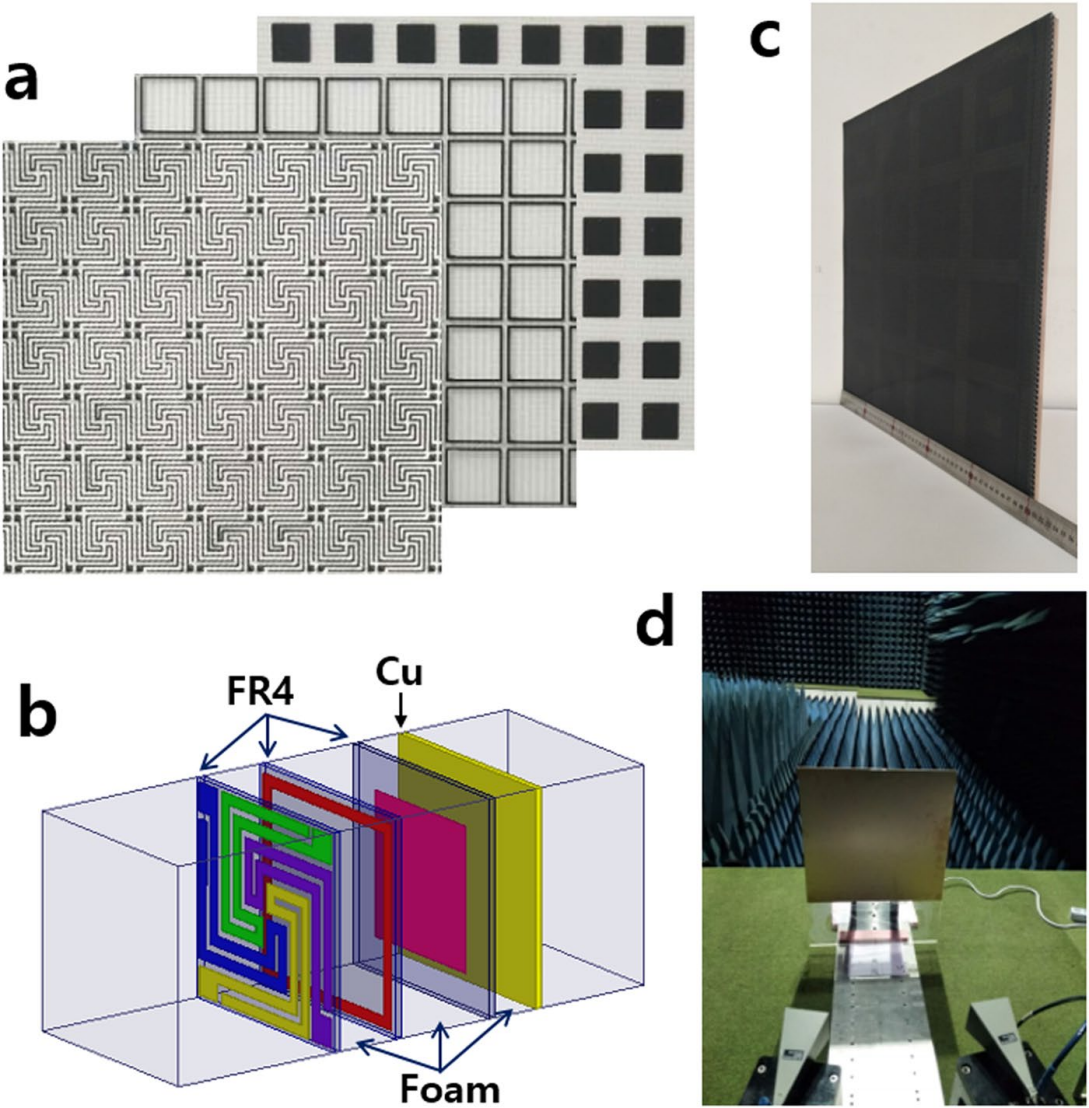
Illustration of test sample and measurement: (**a**) top view of printed patterns of FSS3 (spiral), FSS2 (square loop), FSS1 (square patch) with a unit cell periodicity of 6.0 mm, (**b**) a schematic layer structure, (**c**) a fabricated prototype with 50 cm × 50 cm size and 6.5 mm thickness, and (**d**) free space measurement system in an anechoic chamber.

The three types of FSS (spiral, square loop, square patch) with geometries and surface resistances close to the optimum values (Table 2) were fabricated by a screen printing method. Resistive ink material (carbon black paste) was transferred through a mesh onto a substrate (FR4 substrate with a 0.2 mm thickness) whose printed FSS patterns are presented in Fig. 9a. Using the polyethylene foam as the space material and FR4 as the printing substrates, a three-layer absorber was constructed with a terminal plate of Cu (Fig. 9b). The thickness of the space material was controlled to be t_1_ = 1.8 mm, t_2_ = 2.8 mm, and t_3_ = 1.3 mm. The total thickness was 6.5 mm, including FR4 substrates. The test sample was 50 cm × 50 cm including 83 × 83 unit cell arrays, and the fabricated prototype is shown in Fig. 9c.

**Figure 10:**
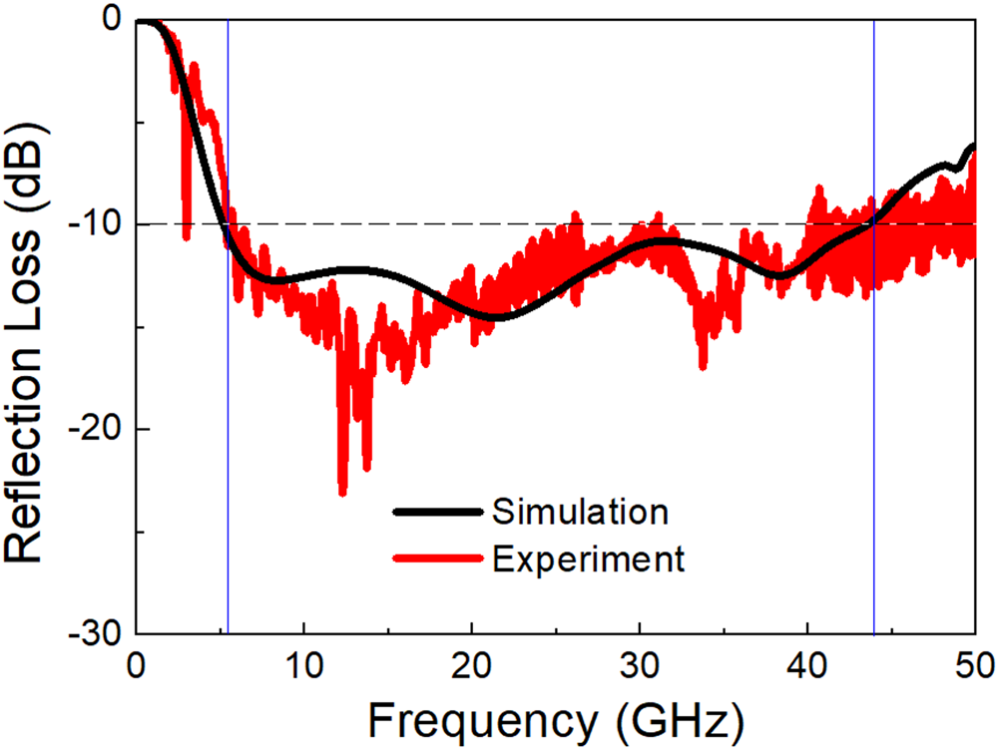
Reflection loss measured by free space test for the triple-layer absorber with a structure presented in Fig. 9b. Simulation result for the same triple-layer structure and surface resistances as the test sample is also given for comparison.

The power reflection loss was measured by the free-space measurement system (Fig. 9d). Figure 10 presents the measurement results of the reflection loss for normal incidence of TEM wave, depicting a 10 dB absorption bandwidth of 5.2–44.0 GHz (FBW = 158%), which is in good agreement with the simulation result for the same multilayer structure and surface resistance of the test sample. Compared with the air substrate, the bandwidth was slightly decreased due to the insertion of high-permittivity materials (FR4) and a slight change in spacer thickness. The experimental results strongly verified the proposed design method.

## Discussions

The most significant result of this study was that a high-capacitive FSS with a new structure of four folded arm spiral conductors could be utilized in the design and fabrication of ultra-wide bandwidth and polarization-insensitive absorbers in a multilayer structure. Due to the folded structure of the spiral FSS with the long effective segments and a small gap, a large value of capacitance for the lowest resonating frequency with a reduced periodicity was obtained. Through a combination of the high-capacitive spiral FSS with other conventional FSSs (square loop, square patch) with a medium- and high-frequency resonance, an ultra-wide absorption bandwidth (4.7–50.0 GHz for −10 dB reflection loss) was designed with a small total thickness of 7.0 mm, which is close to the theoretical limit (6.7 mm). That is the novelty of this study over the existing works from the standpoints of enlarged bandwidth (FBW = 166%) with a comparable thickness (0.11λ_L_) and a simple (three FSS layers) and polarization-insensitive structure of 90° rotation symmetry. We analyzed the possible mechanism from the optimization procedure for the reflection loss and bandwidth by varying the surface resistance of the FSS and the layer thickness of the triple-layer absorber. The free space measurement with a test sample prepared by the screen printing method also demonstrated a wide-bandwidth absorption result (5.2–44.0 GHz (FBW = 158%) for −10 dB reflection loss, total thickness = 6.5 mm (0.12λ_L_)) and was in good agreement with the simulation result. In addition, the angular stability of the proposed wide bandwidth absorber was discussed for both TE and TM polarization in association with unit cell periodicity and grating lobes.

## Methods

Numerical designs and simulations were performed using the commercial software ANSYS-HFSS. Master-slave periodic boundary conditions were applied in the numerical model to mimic a 2D infinite structure. Floquet ports were used for the excitation of the periodic structure. A test sample of 50 cm × 50 cm size including 83 × 83 unit cells array was fabricated and its power absorption was measured. The FSSs with geometries and surface resistances close to the optimum values (Table 2) were fabricated by a screen printing method. Carbon black paste was chosen as the resistive ink, and a commercially available FR4 plate (with permittivity of ε_r_ = 4.05 + j0.017 and 0.2 mm thickness) was used as the printing substrate. The surface resistance of FSS strips was measured by a 4-probe technique. The space material was an open-cell polyethylene foam with a permittivity of ε_r_ = 1.05 + j0.02. Integrating these materials with a terminal plate of Cu, a three-layer absorber was constructed (Fig. 9b). Measurements were carried out in an anechoic chamber using an Agilent vector network analyzer and two horn antennas for four different frequency bands (1–18 GHz, 18–30 GHz, 26.5–40 GHz, 40–50 GHz). After the reflection coefficient was normalized using the backed metallic plate of the structure acting as an ideal reflector, measurements of power reflection were made for the test samples.

## Supplementary information

Supplementary Information

## References

[1] Rappaport TS, MacCartney GR, Mellios E (2017). Overview of millimeter wave communications for fifth-generation (5G) wireless networks—with a focus on propagation models. IEEE Trans. Antennas Propag.

[2] Rozanov KN (2000). Ultimate thickness to bandwidth ratio of radar absorbers. IEEE Trans. Antennas Propag.

[3] Fante RL, McCormack MT, Syst TD, Wilmington MA (1988). Reflection properties of the Salisbury screen. IEEE Trans. Antennas Propag.

[4] David, C. J. *Radar and Laser Cross Section Engineering* 2^nd^ Edition 150–175 (American Institute of Aeronautics and Astronautics, Inc. 2005).

[5] Landy NI, Sajuyigbe S, Mock JJ, Smith DR, Padilla WJ (2008). Perfect metamaterial absorber. Phys. Rev. Lett..

[6] Ra’di Y, Simovski CR, Tretyakov SA (2015). Thin perfect absorbers for electromagnetic waves: theory, design, and realizations. Phys. Rev. Appl..

[7] Watts CM, Liu X, Padilla WJ (2012). Metamaterial electromagnetic wave absorbers. Adv. Mat..

[8] Du Toit LJ (1994). The design of Jauman absorbers. IEEE Antennas Propag. Mag..

[9] Du Toit LJ, Cloete JH (1996). Electric screen Jauman absorber design algorithms. IEEE Trans. Microwave Theory Tech.

[10] Chambers B, Tennant A (1996). Optimized design of Jaumann radar absorbing materials using a genetic algorithm. IEE Proc.-Radar, Sonar Navig.

[11] Costa F, Monorchio A, Manara G (2010). Analysis and design of ultrathin electromagnetic absorbers comprising resistively loaded high impedance surfaces. IEEE Trans. Antennas Propag.

[12] Costa F, Monorchio A (2012). Closed-form analysis of reflection losses in microstrip reflectarray antennas. IEEE Trans. Antennas Propag.

[13] Zhang H-B, Zhou P-H, Lu H-P, Deng L-J (2013). Resistance selection of high impedance surface absorbers for perfect and broadband absorption. IEEE Trans. Antennas Propag.

[14] Pang Y-Q, Zhou Y-J, Wang J (2011). Equivalent circuit method analysis of the influence of frequency selective surface resistance on the frequency response of metamaterial absorbers. J. Appl. Phys..

[15] Sun L, Cheng H, Zhou Y, Wang J (2012). Broadband metamaterial absorber based on coupling resistive frequency selective surface. Opt. Express.

[16] Kazemzadeh A, Karlsson A (2009). Capacitive circuit method for fast and efficient design of wideband radar absorbers. IEEE Trans. Antennas Propag.

[17] Liu T, Kim S-S (2016). Design of wide-bandwidth electromagnetic wave absorbers using the inductance and capacitance of a square loop-frequency selective surface calculated from an equivalent circuit model. Opt. Commnu.

[18] Fallahi A (2010). Thin wideband radar absorbers. IEEE Transactions on Antennas Propag.

[19] Liu S, Chen H, Cui TJ (2015). A broadband terahertz absorber using multi-layer stacked bars. Appl. Phys. Lett..

[20] Peng Y (2015). Ultra-broadband terahertz perfect absorber by exciting multi-order diffractions in a double-layered grating structure. Opt. Express.

[21] Zhu J (2014). Ultra-broadband terahertz metamaterial absorber. Appl. Phys. Lett..

[22] Mou J, Shen Z (2017). Broadband and thin magnetic absorber with non-Foster metasurface for admittance matching. Sci. Rep.

[23] Xiong H (2013). An ultrathin and broadband metamaterial absorber using multi-layer structures. J. Appl. Phys..

[24] Long C (2015). Broadening the absorption bandwidth of metamaterial absorbers by transverse magnetic harmonics of 210 mode. Sci. Rep.

[25] Sun J (2011). An extremely broad band metamaterial absorber based on destructive interference. Opt. Express.

[26] Ghosh S, Bhattacharyya S, Srivastava KV (2016). Design, characterization and fabrication of a broadband polarization-insensitive multi-layer circuit analogue absorber. IET Microwaves, Antennas & Propag.

[27] Li L, Lv Z (2017). Ultra-wideband polarization-insensitive and wide-angle thin absorber based on resistive metasurfaces with three resonant modes. J. Appl. Phys..

[28] Beeharry T (2018). A dual layer broadband radar absorber to minimize electromagnetic interference in radomes. Sci. Rep.

[29] Liu T, Kim S-S (2018). Design of ultra wide-bandwidth double-layer electromagnetic wave absorbers with square-loop frequency selective surfaces. Microw. Opt. Tech. Lett.

[30] Liu T, Kim S-S (2018). Ultrawide Bandwidth Electromagnetic Wave Absorbers Composed of Double-Layer Frequency Selective Surfaces with Different Patterns. Sci. Rep.

[31] Kazemzadeh A (2011). Nonmagnetic ultra wideband absorber with optimal thickness. IEEE Trans. Antennas Propag.

[32] Costa F, Monorchio A, Manara G (2012). Efficient analysis of frequency-selective surfaces by a simple equivalent-circuit model. IEEE Trans. Antennas Propag. Mag.

[33] Parker EA, El Sheikh ANA (1991). Convoluted array elements and reduced size unit cells for frequency-selective surfaces. Proc. Microw. Antennas Propag..

[34] Parker EA, El Sheikh ANA, Lima ACdeC (1993). Convoluted frequency-selective array elements derived from linear and crossed dipoles. Proc. Microw. Antennas Propag..

[35] Simovski CR, Maagt P, Tretyakov SA, Paquay M, Sochava AA (2004). Angular stabilization of resonant frequency of artificial magnetic conductors for TE-incidence. Electron. Lett..

[36] Huang F, Batchelor JC, Parker EA (2006). Interwoven convoluted element frequency selective surfaces with wide bandwidths. Electron. Lett..

[37] Vallecchi A, Schuchinsky AG (2010). Entwined planar spirals for artificial surfaces. IEEE Antennas Wireless Propag. Lett.

[38] Langley RJ, Parker EA (1982). Equivalent circuit model for arrays of square loops. Electron. Lett..

[39] Marcuvitz, N. *Waveguide Handbook* 280–290 (Lightening Source Ltd., 2012).

[40] Liu, T. & Kim, S.-S. High capacitive frequency selective surfaces of folded spiral conductor arrays. *Microw. Opt. Technol. Lett*., 10.1002/mop.32006 1–7 (2019).

[41] Wadell, B. C. *Transmission Line Design Handbook* 380–386 (Artech House, 1991).

[42] Chen Q (2015). A miniaturized absorptive frequency selective surface. IEEE Trans. Antennas Propag. Lett.

