# Supplementary material for: Ultrawide Bandwidth Electromagnetic Wave Absorbers Using a High-capacitive Folded Spiral Frequency Selective Surface in a Multilayer Structure
Source: Sci Rep. 2019 Nov 11;9:16494. doi: 10.1038/s41598-019-52967-z (PMC6848185; doi:10.1038/s41598-019-52967-z)
Supplement: Supplementary file 1 — Supplementary Information [file 41598_2019_52967_MOESM1_ESM.pdf]

Ultrawide Bandwidth Electromagnetic Wave Absorbers Using a High-capacitive Folded Spiral Frequency Selective Surface in a Multilayer Structure

Tian Liu and Sung-Soo Kim*

Department of Advanced Materials Engineering, Chungbuk National University, Cheongju 361-763, Korea

*Correspondence and requests for materials should be addressed to S.K. (email: sskim@chungbuk.ac.kr)

Table S1. Circuit resistance (*R*) of FSSs with an increasing surface resistance (R_s).

R_s (Ω/sq)	20	100	200	300
R (Ω) for S-FSS	64	320	638	957
R (Ω) for SL-FSS	214	1071	2143	3214
R (Ω) for SP-FSS	45	225	450	675

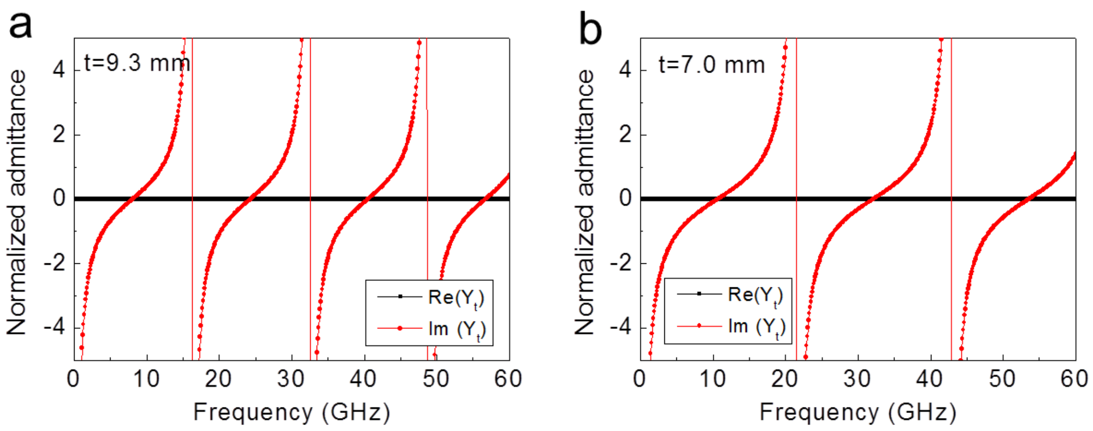

Figure S1. Normalized admittance (Y_t/Y_0) of the grounded substrate with a thickness (t): (a) t = 9.3 mm and (b) t = 7.0 mm.
